# Adult Memory T Cell Responses to the Respiratory Syncytial Virus Fusion Protein During a Single RSV Season (2018–2019)

**DOI:** 10.3389/fimmu.2022.823652

**Published:** 2022-03-29

**Authors:** Brittani N. Blunck, Laura S. Angelo, David Henke, Vasanthi Avadhanula, Matthew Cusick, Laura Ferlic-Stark, Lynn Zechiedrich, Brian E. Gilbert, Pedro A. Piedra

**Affiliations:** ^1^Department of Molecular Virology and Microbiology, Baylor College of Medicine, Houston, TX, United States; ^2^Department of Pathology, University of Michigan, Ann Arbor, MI, United States; ^3^Verna and Marrs McLean Department of Biochemistry and Molecular Biology, Baylor College of Medicine, Houston, TX, United States; ^4^Department of Pharmacology and Chemical Biology, Baylor College of Medicine, Houston, TX, United States; ^5^Department of Pediatrics, Baylor College of Medicine, Houston, TX, United States

**Keywords:** respiratory syncytial virus (RSV), infection, fusion protein, peptide library, memory T cell, polyfunctionality, viral immunity

## Abstract

Respiratory Syncytial Virus (RSV) is ubiquitous and re-infection with both subtypes (RSV/A and RSV/B) is common. The fusion (F) protein of RSV is antigenically conserved, induces neutralizing antibodies, and is a primary target of vaccine development. Insight into the breadth and durability of RSV-specific adaptive immune response, particularly to the F protein, may shed light on susceptibility to re-infection. We prospectively enrolled healthy adult subjects (*n* = 19) and collected serum and peripheral blood mononuclear cells (PBMCs) during the 2018–2019 RSV season. Previously, we described their RSV-specific antibody responses and identified three distinct antibody kinetic profiles associated with infection status: uninfected (*n* = 12), acutely infected (*n* = 4), and recently infected (*n* = 3). In this study, we measured the longevity of RSV-specific memory T cell responses to the F protein following natural RSV infection. We stimulated PBMCs with overlapping 15-mer peptide libraries spanning the F protein derived from either RSV/A or RSV/B and found that memory T cell responses mimic the antibody responses for all three groups. The uninfected group had stable, robust memory T cell responses and polyfunctionality. The acutely infected group had reduced polyfunctionality of memory T cell response at enrollment compared to the uninfected group, but these returned to comparable levels by end-of-season. The recently infected group, who were unable to maintain high levels of RSV-specific antibody following infection, similarly had decreased memory T cell responses and polyfunctionality during the RSV season. We observed subtype-specific differences in memory T cell responses and polyfunctionality, with RSV/A stimulating stronger memory T cell responses with higher polyfunctionality even though RSV/B was the dominant subtype in circulation. A subset of individuals demonstrated an overall deficiency in the generation of a durable RSV-specific adaptive immune response. Because memory T cell polyfunctionality may be associated with protection against re-infection, this latter group would likely be at greater risk of re-infection. Overall, these results expand our understanding of the longevity of the adaptive immune response to the RSV fusion protein and should be considered in future vaccine development efforts.

## Introduction

RSV is a major global health burden as it is a leading cause of acute lower respiratory infection (ALRI) in young children and the elderly (1). RSV causes approximately 22% of all severe ALRI worldwide resulting in over 30 million annual cases and 3 million hospitalizations. These hospitalizations result in 55,000–199,000 deaths, 50,000–75,000 of which are in-hospital deaths in children under the age of 5 years (1, 2). In addition to infants and young children, RSV causes significant morbidity and mortality in older adults and immunocompromised individuals, with a similar disease burden to influenza (3–6).

Immune responses to the initial and subsequent RSV exposures are non-sterilizing, as evidenced by re-infection throughout life (6, 7). This inadequate immune response is not caused by the viral evasion of the immune system seen with other respiratory viruses, including influenza, and is most notable in human challenge studies showing that individuals can be re-infected within two months with identical viral inoculum (7). Why or how the primary immune response fails to protect from subsequent RSV exposure remains unclear. RSV-specific serum antibody, particularly neutralizing antibody, increases protection against re-infection and reduces severe disease in young children, young adults, and the elderly (8–13). Maternal-infant cord blood demonstrates that neutralizing activity correlates with protection of infants from severe disease (14). However, older adults hospitalized with RSV have levels of neutralizing antibody that are considered protective in young children (15), implying that either their repertoire of neutralizing antibodies are less effective or there are other more critical mediators of protection in this population. Therefore, the pathogenesis of disease in re-infection in older adults is likely to require immune mechanisms of protection that are different from that required for the initial infection in infants and young children. Fatal infant cases of RSV demonstrate an almost complete absence of T cells and NK cells in the lungs, illustrating a critical role for these immune cells in controlling viral replication and clearance (16). Conversely, T cells have been implicated in the disease pathogenesis of RSV by causing rampant inflammation (17–19). The longevity and durability of the T cell response following natural RSV-infection in RSV-primed individuals and its role in providing protection from re-infection or severe disease remains unclear.

The F protein, which mediates fusion between the viral and host cell membranes, is the primary focus of the neutralizing antibody response (20). It is also largely conserved between the two subtypes, RSV/A and RSV/B (21), making it a primary target of vaccination efforts (22). An enhanced understanding of the range and longevity of the RSV-specific adaptive immune response, particularly to the F protein, may shed light on the susceptibility to re-infection throughout life. In this study, we evaluated the RSV-specific memory T cell responses to the F protein in healthy adult subjects over the course of a single RSV season and found that memory T cell responses followed the three distinct antibody kinetic profiles that are associated with their RSV infection status: uninfected, acutely infected, and recently infected (23).

## Materials and Methods

### Study Design

Healthy adults were eligible for enrollment into a longitudinal prospective study during the 2018–2019 RSV season in Houston, Texas, United States, as described previously (23). The Institutional Review Board at Baylor College of Medicine approved the study protocol prior to initiation of the study. Written informed consent was obtained from all enrolled participants prior to any study related procedures. Briefly, nineteen healthy adults were enrolled and completed the study. Blood samples were collected at three time points (Visits 1, 2, and 3), which occurred in November 2018, January 2019, and May 2019, respectively. RSV infection status was determined by changes in RSV neutralizing antibody titers using four qualified microneutralization assays (24) utilizing prototypic (RSV/A/Tracy and RSV/B/18537) and contemporaneous (RSV/A/Ontario and RSV/B/Buenos Aires) isolates. Volunteers with less than a four-fold change in RSV neutralizing antibody activity over the course of the season by all four assays were defined as uninfected; those with four-fold or greater increases between two consecutive study visits by one or more assay were defined as having an acute RSV infection; and those with a four-fold or greater decrease in neutralizing antibody titer at their second visit by one or more assay were defined as having a recent infection prior to enrollment, indicating we missed the baseline titer prior to RSV infection (23).

### Peripheral Blood Mononuclear Cell Isolation

Blood was collected in sodium citrate CPT tubes (BD Vacutainer, Cat. #62761) and processed within four hours of collection. PBMCs were isolated by centrifugation for 30 minutes at 1800 x g (RCF) at room temperature (21°C). Cells were washed 3 times in phosphate buffered saline (PBS) with centrifugation at 300 x g (RCF) for 10 minutes at room temperature (21°C). Cells were frozen in 10% dimethyl sulfoxide (DMSO) in fetal bovine serum (FBS) and stored in liquid nitrogen.

### Fusion Protein Peptide Library Generation

Overlapping peptide libraries of the full-length RSV F0 protein derived from RSV/A/Ontario (GenBank ID: AZQ19478.1) was custom ordered (Genentech, San Francisco, CA) and RSV/B/B1 (Swiss-Prot ID: O36634) was obtained from JPT (Berlin, Cat. #PM-HRSVB-FGF0). Each library contained 141 15-mer peptides with an 11 amino acid overlap (25, 26). Each peptide library was reconstituted in DMSO and stored at -80°C in single use aliquots.

### *In Vitro* Stimulation and Multiparametric Flow Cytometry

PBMCs were rapidly thawed in a 37°C water bath and added dropwise into pre-warmed R10 medium (RPMI 1640 + 10% FBS). Cells were washed in R10 medium to remove excess DMSO, and viable cells were counted using trypan blue exclusion. Cells were resuspended at 1.5 x 10^6^ cells/mL in 5 mL of R10 medium in 50 mL conical tubes and rested overnight at 37°C in 5% CO_2_. Tubes were placed at a 5° angle, and the cap loosened to allow for maximum oxygenation (27). After resting overnight, samples were plated into 96-well round bottom plates. Cells were stimulated with either R10 medium alone (negative unstimulated control), PMA/ionomycin (positive control), the RSV/A/Ontario F (RSV F_A_) protein peptide library, or the RSV/B/B1 F (RSV F_B_) protein peptide library. Both RSV F_A_ and RSV F_B_ protein peptide library contained anti-CD28 and anti-CD49d co-stimulatory agents (Becton-Dickinson Biosciences, Franklin Lakes, NJ Cat. #347690) with brefeldin A, monensin, and anti-CD107a antibody. Stimulation was for 6 hours at 37°C in 5% CO_2_ (28, 29). Following stimulation, cells were washed in PBS (without Ca^++^ or Mg^++^), and viability dye (ThermoFisher Scientific, Waltham, NJ) was added to enable gating out any non-viable cells. Fc-blocking was performed to reduce non-specific binding of antibodies using 5% FBS in PBS. Extracellular antibodies were then added and incubated for 20 minutes in the dark at room temperature. Following washing, cells were fixed and permeabilized (BD Cyto Fixation/Permeabilization kit, Cat #554714) for 20 minutes in the dark at 4°C. Cells were washed twice with BD CytoWash solution (BD Cyto Fixation/Permeabilization kit, Cat #554714). Antibodies for intracellular markers were then added for intracellular staining and incubated for 30 minutes in the dark at 4°C. Cells were washed twice in BD CytoWash solution and then cells were resuspended in 1% paraformaldehyde prior to acquisition. In total, samples were stained with a pool of fluorescence-conjugated antibodies for CD45, CD56, CD16, CD3, CD4, CD8, CD45RO, CD107a, TNFα, IFNγ, and PD-1. Cells were analyzed on an LSRII-Fortessa flow cytometer running DiVa software (Becton-Dickinson Biosciences, Franklin Lakes, NJ), and data were analyzed using FlowJo (version 10.7.1; TreeStar, OR) and Simplified Presentation of Incredibly Complex Evaluations (SPICE; National Institute of Health, Bethesda, MD) software. Viable lymphocytes were identified by forward- and side-scatter, single-cell discrimination, live/dead measurements using viability dye exclusion, and expression of the pan-lymphocyte marker CD45.

### Uniform Manifold Approximation and Projection Visualization of Flow Cytometric Data

Contour plots were generated using ‘contour’ visualization in FlowJo (using equal probability contouring). For uniform manifold approximation and projection (UMAP) analysis, all samples were down-sampled to 5,000 cells using the DownSample plugin (v3.3) available on FlowJo Exchange. All samples were concatenated to create a single, 1,140,000 cell composite, and a UMAP algorithm for dimensionality reduction was applied using the UMAP plugin (v3.1) available on FlowJo Exchange (30, 31). The composite sample was then re-gated as indicated for all primary and secondary populations to aid in visual overlays in exploration of the UMAP projections. Density plots representing 90% of the total gated cells by RSV infection status or stimulation were superimposed upon UMAP projections to visualize differences by study visit.

### Simplified Presentation of Incredibly Complex Evaluation Analysis

Simplified Presentation of Incredibly Complex Evaluation (SPICE) is a software that can be used to analyze multivariate data sets for which a series of nominal measurements and a single continuous measurement is available. We employed SPICE software in our study as a means to visually inspect and represent the polyfunctionality of T cell subsets in response to stimulation with either RSV F_A_ or RSV F_B_ protein peptide libraries (32). SPICE analysis is largely qualitative rather than quantitative and is used to provide an overall commentary of the trends in the data. No statistical conclusions were drawn from the SPICE data and we do not refer to any differences in polyfunctional responses as “significant” since other methods were used to determine statistical significance throughout the manuscript.

### High Resolution Human Leukocyte Antigen-Typing

Blood was collected in Acid Citrate Dextrose tubes (Becton-Dickinson Biosciences, Franklin Lakes, NJ) and DNA was extracted from whole blood using the Qiagen EZ1^®^ DNA Blood 350 µl Kit (Qiagen, Hilden, Germany) with the EZ1 Advanced system. After extraction, DNA concentration and quality were measured with the Qiagen Qiexpert spectrophotometer. Next generation sequencing (NGS) human leukocyte antigen (HLA)-typing for HLA-A, -B, -C, -DRB1, -DRB3/4/5, -DQB1, -DQA1, -DPB1, and -DPA1 was done using MIA FORA kit (Immucor, Norcross, GA), according to the manufacturer’s instructions. Briefly, after long-range PCR amplification of each HLA gene, DNA fragments (500–900 bp) were selected, amplified, cleaned, and sequenced on a MiSeq using MiSeq Reagent Kits v2 (300 cycles) (Illumina, San Diego, CA). Samples were analyzed using MIA FORA NGS software.

### Prediction of RSV/A and RSV/B F Protein T Cell Epitopes

T cell epitopes within the RSV F_A_ and RSV F_B_ protein peptide libraries were predicted using the Immune Epitope Database and Analysis Resource (IEDB, National Institute of Allergy and Infectious Disease, Bethesda, MD) major histocompatibility complex (MHC)-I and MHC-II binding algorithms (33, 34). Only HLA alleles from our cohort were included in the predictions and allele-specific percentile ranks of all algorithms queried by the IEDB tool were utilized (35). A percentile rank is generated by comparing the predicted binding affinity of a selected peptide against that of a large set of similarly sized peptides randomly selected from the SWISS-PROT database (36). Percentile rank provides a uniform scale allowing comparisons across different predictors. A lower percentile rank indicates higher affinity. Predicted hits were further refined to those specifically within our peptide libraries utilizing a threshold of <5% for both MHC-I and MHC-II.

### Statistical Analysis

A repeated measures mixed model analysis was performed to assess differences in expression of each functional marker among the three RSV infection status groups and three study visits. The covariance structure and diagnostic plots of the residuals were examined to assess the validity of the model assumptions for a repeated measures analysis of variance approach. The analysis first determined whether the visit by infection status interaction term in the model was significant by the omnibus F-test. Pairwise comparisons were conducted only of the mean percentage difference between the visits within each infection status group for a total of nine *a priori* comparisons per functional marker. Statistical significance was indicated for *P* values ≤ 0.05. No correction was made for multiple comparisons. T cell and neutralizing antibody scores were calculated by quartile ranking responses, where the top quartile received a score of 4 and the lowest quartile received a score of 1. Populations of T cells that received scores included: total T cells, CD4^+^ memory T cells, CD8^+^ memory T cells and CD4^+^/CD8^+^ memory T cells which were summed for each individual to create a composite score with a range of 4–16. Separate T cell composite scores were calculated for responses to the RSV F_A_ and RSV F_B_ protein peptide libraries. Neutralizing antibody score was calculated by quartile ranking neutralizing antibody titers to RSV/A/Tracy, RSV/B/18537, RSV/A/Ontario, and RSV/B/Buenos Aires which were summed for each individual to create a composite score with a range of 4–16. Pearson’s correlation coefficients were calculated between T cell scores to each peptide library and corresponding neutralizing antibody scores. Statistical analyses were performed using Stata 14 (Stata Corp, College Station, Texas).

## Results

### Demographics

Healthy adults under the age of 65 with no underlying conditions were enrolled during the 2018–2019 RSV season, where RSV/B was the dominant circulating subtype, as described previously (23). There were three RSV infection status groups, which were defined by changes in neutralizing antibody titer: uninfected (n=12), acutely infected (n=4), and recently infected (n=3). Volunteers with less than a four-fold change in RSV neutralizing antibody activity over the course of the season by all four assays were defined as uninfected; those with four-fold or greater increases between two consecutive study visits by one or more assay were defined as having an acute RSV infection; and those with a four-fold or greater decrease in neutralizing antibody titer at their second visit by one or more assay were defined as having a recent infection prior to enrollment, indicating we missed the baseline titer prior to RSV infection. Ages ranged from 23–59, with no discernable difference detected among age, gender, or ethnicity across infection status (23).

### Total T Cell Responses to RSV F Protein Peptide Libraries

**Figure 1:**
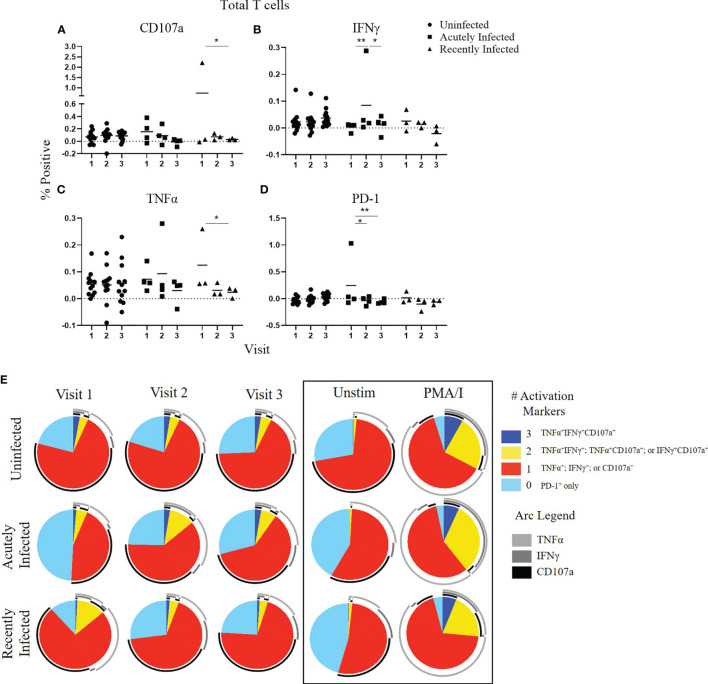
Total T cell responses to the RSV F protein peptide libraries as a function of RSV infection status and study visit. **(A–D)** Individual functional marker expression following stimulation with the RSV/A F (RSV F_A_) protein peptide library by RSV infection status: uninfected (*n* = 12), acutely infected (*n* = 4), and recently infected (*n* = 3). PBMCs from adult volunteers were stimulated with the RSV F_A_ protein peptide library and the expression of CD107a, IFNγ, TNFα, and PD-1 were measured relative to the unstimulated controls by flow cytometry. These values are reported as percent positive of total CD3^+^ T cells. Each symbol represents the response from a single individual. The thick horizontal bar indicates the mean of all responses within each group at that visit. A significant pairwise comparison of mean percentage difference between visits within a group is denoted by a thin horizontal bar with *P ≤ 0.05, **P ≤ 0.01. **(E)** Polyfunctional T cell responses to RSV F_A_ protein peptide library by RSV infection status. Simplified Presentation of Incredibly Complex Evaluations (SPICE) software was used for the identification of total T cells expressing the various activation markers. Pie charts show the frequency in which PBMCs produced the various combinations of the activation markers CD107a, IFNγ, and TNFα; or expressed PD-1 alone. Background (determined from the media-stimulated negative controls) was subtracted from all samples and negative values were set to zero. Surrounding arcs denote the specific markers produced by the cells in each pie segment. Representative negative and positive controls across all study visits are boxed.

**Figure 2:**
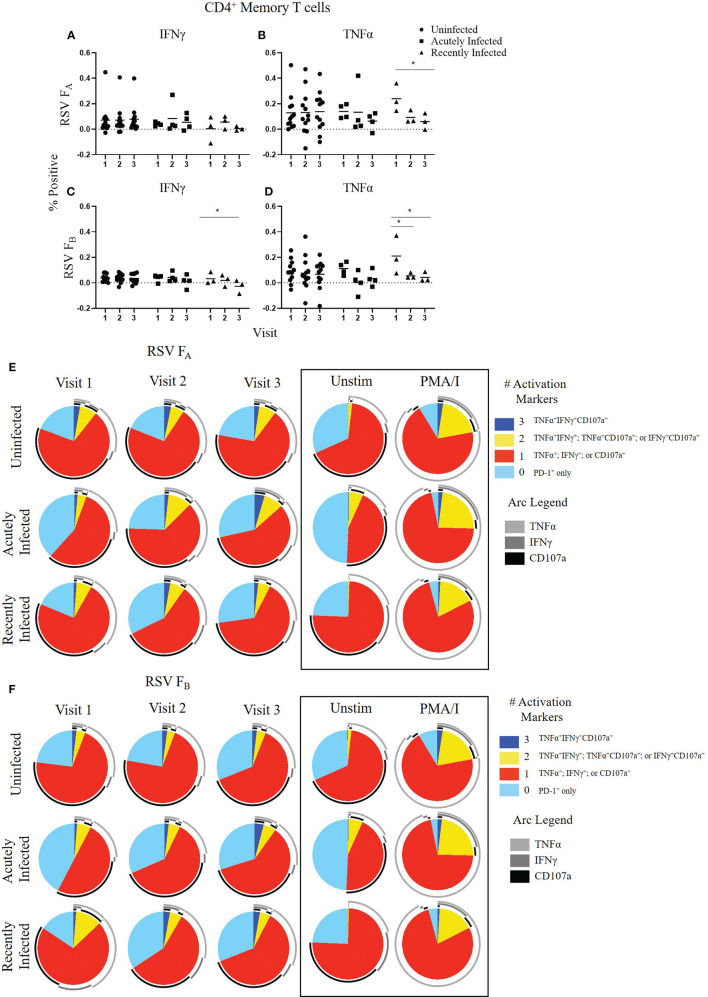
CD4^+^ Memory T cell responses to the RSV F protein peptide libraries by RSV infection status and study visit. **(A–D)** Individual functional marker expression by RSV infection status: uninfected (*n* = 12), acutely infected (*n* = 4), and recently infected (*n* = 3). PBMCs were stimulated *in vitro* with RSV F_A_** (A, B) **or RSV F_B_** (C, D) **F protein peptide libraries. Marker expression is shown as percentage of total CD4^+^ memory T cells. symbol represents the response from a single individual. The thick horizontal bar indicates the mean of all responses within each group at that visit. A significant pairwise comparison of mean percentage difference between visits within a group is denoted by a thin horizontal bar with *P ≤ 0.05. **(E, F)** Polyfunctional CD4^+^ memory T cell responses to RSV F_A_ or RSV F_B_ protein peptide libraries as a function of RSV infection status and study visit. Simplified Presentation of Incredibly Complex Evaluations (SPICE) analysis was performed for the identification of CD4^+^ memory T cells expressing multiple activation markers. Pie charts show the frequency in which PBMCs produced the various combinations of the activation markers CD107a, IFNγ, and TNFα; or expressed PD-1 alone. Background (determined from the media-stimulated negative controls) was subtracted from all samples and negative values were set to zero. Surrounding arcs denote the specific markers produced by the cells in each pie segment. Representative negative and positive controls across all study visits are boxed.

To compare functional responses of T cells among the three infection status groups, we first analyzed the total T cell response (CD3^+^,CD56^-^; **Figure 1**) by measuring the expression of four functional markers: CD107a, IFNγ, TNFα, and PD-1 using the antibody panel shown in **Supplementary Table 1**. A representative gating strategy is shown in **Supplementary Figure 1A**. All gates were set from fluorescence minus one (FMO) controls (**Supplementary Figure 1B**). CD107a (also known as LAMP-1) is a marker of degranulation of cytolytic T cells, whereas IFNγ and TNFα are pro-inflammatory cytokines, and PD-1 is a surface protein that negatively regulates immune responses, which serves as a marker of T cell exhaustion (37–39). In addition to analyzing single expression of these four markers, we also analyzed the polyfunctionality of the T cell response since the magnitude of a T-cell response as measured by a single parameter does not fully reflect its functional potential (40). Higher polyfunctionality can indicate a higher quality anti-viral immune response and is often used to evaluate the quality of vaccine-induced immune responses. Several studies have provided compelling evidence that the quality of the T cell response is a crucial factor in defining a protective T cell response (40–48). Consistent with the stability of their neutralizing antibody response (23), the uninfected group had a stable total T cell response over the course of the RSV season as measured by either single functional marker expression (**Figures 1A–D**) or polyfunctionality (as defined by dark blue and yellow pie slices) of activation markers CD107a, IFNγ, and TNFα (**Figure 1E**). The acutely infected group had minimal changes in single activation marker expression throughout the season but had significantly higher PD-1 expression at enrollment (Visit 1; **Figures 1A–E**). The T cells from the acutely infected group also displayed less polyfunctionality at enrollment compared to the uninfected group but regained polyfunctionality, comparable to the levels in the uninfected group by Visit 3. This pattern is similar to that observed with the neutralizing antibody responses for the acutely infected and uninfected groups (**Figure 1E**) (23). The total T cell response of the recently infected group followed a pattern similar to their neutralizing antibody response. There was a significant decline in CD107a and TNFα expression over the course of the season (**Figures 2A, C**), as well as a reduction of polyfunctionality, although polyfunctionality remained low in comparison to the uninfected group at Visit 3 (**Figure 2E**). Total polyfunctional profiles with combinations of all four functional markers (CD107a, IFNγ, TNFα, and PD-1) followed similar patterns as described for the polyfunctional profiles of the three activation markers (**Supplementary Figure 2**). Additionally, all trends observed with the RSV F_A_ protein peptide library were also observed following stimulation with the RSV F_B_ F protein peptide library (data not shown).

### Uniform Manifold Approximation and Projection Analysis

We next wanted to consider whether there were global differences in T cells by RSV infection status or by RSV subtype. To aid in exploration of the dataset, we created a composite sample by representative down-sampling (5,000 cells per sample) flow cytometry results obtained from each study subject at each study visit using the DownSample plugin (v3.3) available on FlowJo Exchange. A sample UMAP algorithm for dimensionality reduction was applied to gated live T lymphocytes (CD45^+^CD3^+^CD56^-^) composite sample and assessed all additional fluorescence markers (**Supplementary Figure 3**). Cells from the composite sample were mapped in Cartesian space (**Supplementary Figure 3A**). Gating on T cell subsets following the strategy outlined in **Supplementary Figure 1**, confirmed that UMAP analysis clustered distinct cell phenotypes (**Supplementary Figure 3B**). We compared UMAP clustering among infection status groups to understand whether there are global differences in T cell populations in response to stimulation with the RSV F protein peptide libraries that could explain, at least in part, the different antibody kinetic profiles of our cohort (**Supplementary Figure 3C**).

We found that whereas there were variations within each infection status group at the various study visits, there were no variations associated with infection status (**Supplementary Figure 3C**). To assess whether there were RSV subtype-specific differences, we compared T cell responses following stimulation with the two different RSV F protein peptide libraries (**Supplementary Figure 3D**) and found that there was no difference. Virus-specific T cell responses are rare events. Therefore, these results were not surprising and indicate that any differences in responses among the subject groups or RSV virus-subtypes were based upon functionality rather than broad T cell phenotype.

### CD4^+^ Memory T Cell Responses to RSV F Protein Peptide Libraries

We evaluated CD4^+^ memory T cell (CD45RO^+^CD4^+^ T cells) responses (**Figure 2** and **Supplementary Figure 4**). CD107a and PD-1 expression did not change significantly within any of the groups following stimulation with either the RSV F_A_ or RSV F_B_ protein peptide library and are therefore not shown. As observed with the total T cell response, the uninfected group had a very stable CD4^+^ memory T cell response to both F protein peptide libraries over the course of the RSV season as measured by single functional marker expression (**Figures 2A–D**) or by polyfunctionality of the activation markers (**Figures 2E, F**). The acutely infected group also had a very stable CD4^+^ memory T cell response by single functional marker analysis (**Figures 2A–D**), but polyfunctional analyses revealed a distinct profile to that of the uninfected group with changes in polyfunctionality over the study duration when stimulated with either RSV F_A_ or RSV F_B_ protein peptide libraries (**Figures 2E, F**). The acutely infected group had a polyfunctional profile driven by increased PD-1 expression and reduced polyfunctionality compared to the uninfected group at enrollment (Visit 1). Polyfunctionality increased over the RSV season in the acutely infected group and more closely resembles that of the uninfected group by Visit 3. The recently infected group had significant decreases in CD4^+^ memory T cell single marker expression of IFNγ, and TNFα over the course of the season but had only subtle changes in polyfunctionality over the RSV season (**Figures 2B, C**). The recently infected group had an increased double functionality (co-expression of two activation markers, indicated by yellow pie slice) and reduced triple functionality (co-expression of all 3 activation markers, indicated by dark blue) compared to the uninfected group at Visit 1. By Visit 3, however, the polyfunctional profile of CD4^+^ memory T cells was comparable to that of the uninfected group.

### CD8^+^ Memory T Cell Responses to RSV F Protein Peptide Libraries

**Figure 3:**
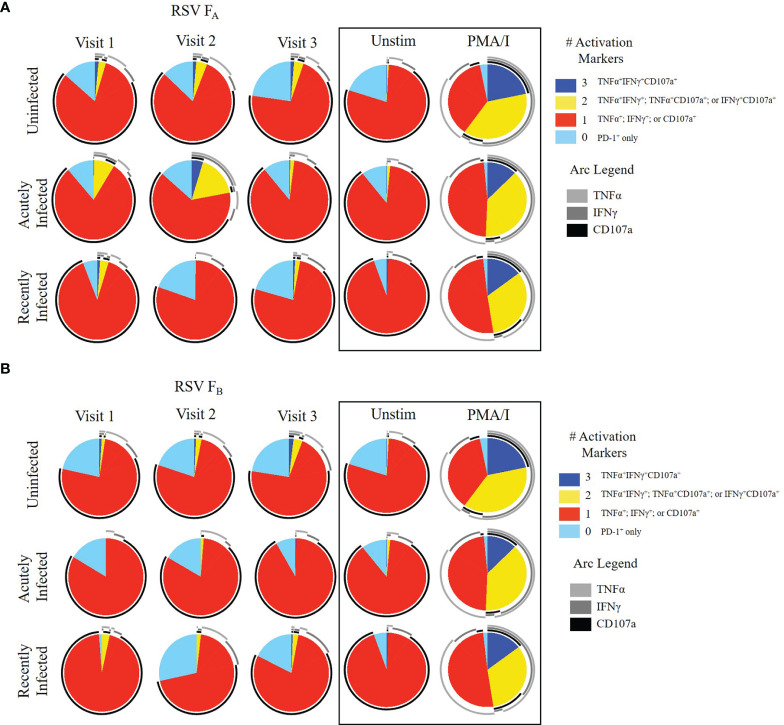
CD8^+^ Memory T cell responses to RSV F protein peptide libraries as a function of RSV infection status and study visit. Polyfunctional CD8^+^ Memory T cell responses to **(A)** RSV F_A_ or **(B)** RSV F_B_ protein peptide libraries by RSV infection status: uninfected (*n* = 12), acutely infected (*n* = 4), and recently infected (*n* = 3). Simplified Presentation of Incredibly Complex Evaluations (SPICE) analysis was performed for the identification of CD8^+^ memory T cells expressing multiple cytokines. Pie charts show the frequency in which PBMCs produced the various combinations of the activation markers CD107a, IFNγ, and TNFα; or expressed PD-1 alone. Background (determined from the media-stimulated negative controls) was subtracted from all samples and negative values were set to zero. Surrounding arcs denote the specific markers produced by the cells in each pie segment. Representative negative and positive controls across all study visits are boxed.

We then evaluated CD8^+^ memory T cell (CD45RO^+^CD8^+^ T cells) responses (**Figure 3**, and **Supplementary Figures 5**, **6**). The expression of single functional markers by CD8^+^ memory T cells remained the same for each infection status for the duration of the study (**Supplementary Figure 5**). Although the expression of single functional markers was both low and stable, the polyfunctional profiles were distinct among the three groups (**Figure 3** and **Supplementary Figure 6**). Like the other subsets, the polyfunctionality of the CD8^+^ memory T cell response in the uninfected group was consistent across the three study visits to both RSV F protein peptide libraries (**Figures 3A, B**). In the acutely infected group, there was a lack of triple functionality toward the RSV F_A_ protein peptide library at enrollment (Visit 1; **Figure 3A**). Polyfunctionality in the acutely infected group expanded following infection at Visit 2 but retracted by Visit 3 to levels slightly lower than the uninfected group. Polyfunctionality of CD8^+^ memory T cells toward the RSV F_B_ protein peptide library in the acutely infected group was nearly absent over the study period (**Figure 3B**). The CD8^+^ memory T cell response of the recently infected group was distinct from that of the uninfected or acutely infected group and displayed very little polyfunctionality toward either peptide library. This distinct profile is quite notable, particularly at Visit 1, when combinations of all four functional markers were analyzed (**Supplementary Figure 6**).

### CD4^+^/CD8^+^ Memory T Cell Responses to RSV F Protein Peptide Libraries

CD4^+^/CD8^+^ double positive T cells make up a low frequency of total T cells and can express memory markers such as CD45RO. Their role in viral immunity and cancer is hotly debated, though there is evidence they may have enhanced anti-viral capabilities (49–52). We therefore analyzed the CD4^+^/CD8^+^ memory T cell response among the infection status groups and found that the trends mimic those of the canonical CD4^+^ or CD8^+^ memory T cell response (**Supplementary Figure 7**). Similar to the other subsets of T cells, the uninfected group had very consistent levels of CD4^+^/CD8^+^ memory T cell response over the RSV season to both RSV F protein peptide libraries. The acutely infected group had a significant decrease in TNFα expression following infection, but by Visit 3 TNFα expression had returned to levels observed at enrollment. The recently infected group had a significant decrease in CD107a expression over the RSV season. Taken altogether, responses of all T cell subsets, as measured by both the magnitude of single parameters and polyfunctionality, closely mimics that observed in the antibody kinetic profiles by RSV infection status (23).

### Subtype-Specific Differences in T Cell Responses to RSV F Protein Peptide Libraries

**Figure 4:**
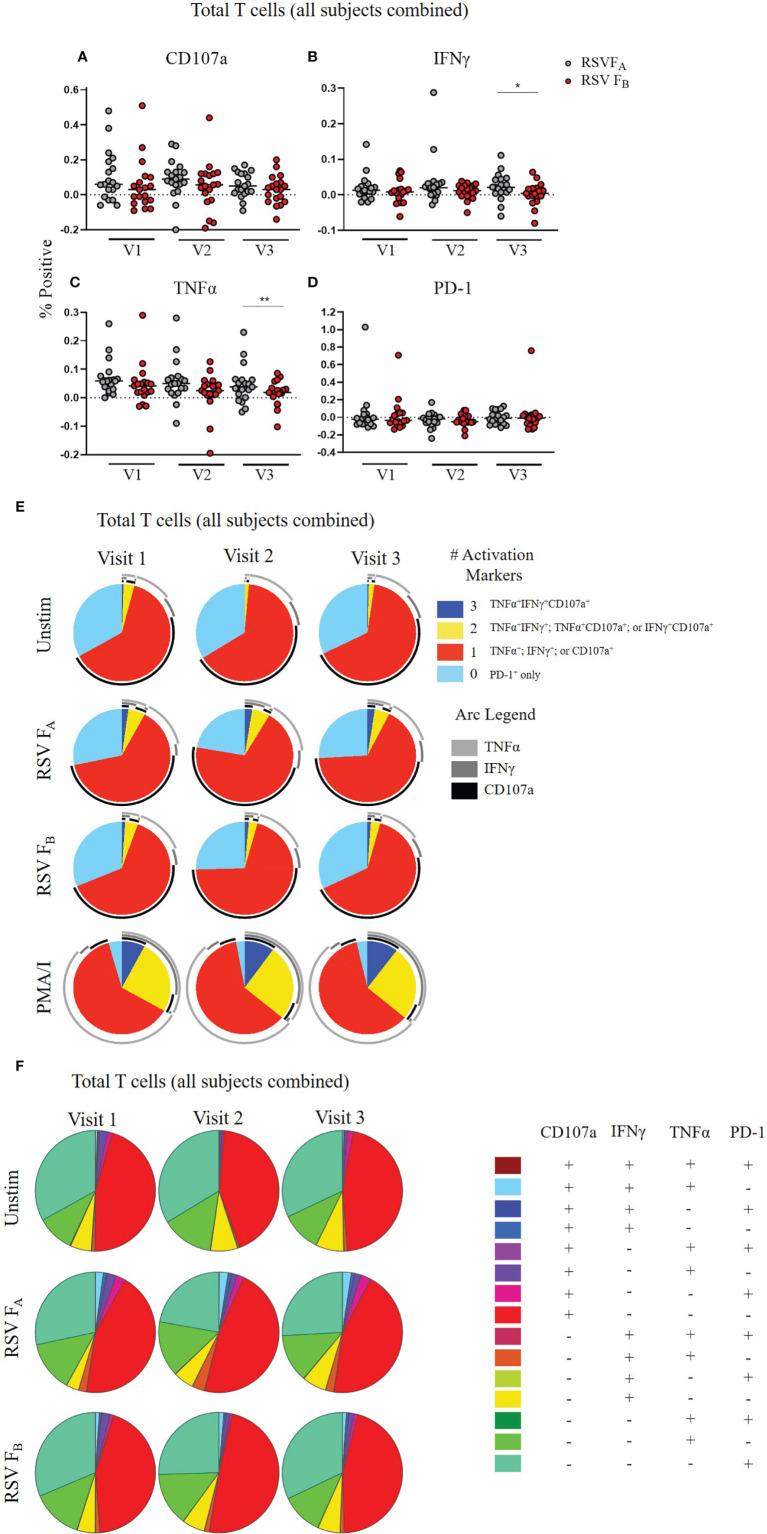
RSV subtype-specific differences in total T cell responses to RSV F protein peptide libraries for all study subjects. **(A–D)** Individual functional marker expression following stimulation with either peptide library (*n* = 19). PBMCs were stimulated with either RSV F_A_ or RSV F_B_ peptide library and expression of CD107a, IFNγ, TNFα, and PD-1 were measured by flow cytometry and reported as a percentage of total T cells. Each symbol represents the response from a single individual. The thick horizontal bar indicates the mean of all responses within each group at that visit. A significant pairwise comparison of mean percentage difference between visits within a group is denoted by a thin horizontal bar with *P ≤ 0.05, **P ≤ 0.01. V1, Visit 1; V2, Visit 2; V3, Visit 3. **(E)** Polyfunctionality of activation markers in total T cell responses as a function of stimulation type and study visit. Pie charts show the frequency in which PBMCs produced the various combinations of the activation markers CD107a, IFNγ, and TNFα; or expressed PD-1 alone. Background (determined from the media-stimulated negative controls) was subtracted from all samples and negative values were set to zero. Surrounding arcs denote the specific markers produced by the cells in each pie segment. Representative negative and positive controls across all study visits are boxed. **(F)** Total polyfunctionality of total T cells by stimulation and study visit. Pie segments indicate frequency of cells producing combinations of all four functional markers CD107a, IFNγ, and TNFα and PD-1. Background (determined from the media-only negative controls) was subtracted from all samples and negative values were set to zero.

Because we observed differences between the responses to the RSV F_A_ and RSV F_B_ protein peptide libraries across all infection groups and T cell subsets, we combined these data for all 19 adults at each timepoint to assess viral subtype-specific differences in T cell responses. In total T cells, the RSV F_A_ protein peptide library induced higher expression of each individual activation marker (CD107a, IFNγ, and TNFα) than the RSV F_B_ protein peptide library (**Figures 4A–D**). Whereas this trend is only significant at Visit 3 for IFNγ and TNFα expression, the trend was consistent at every timepoint for these three activation markers. PD-1 expression, however, was similar between the two peptide libraries, indicating that stimulation with the RSV F_B_ peptide library is not simply exhausting the T cells. There was also decreased polyfunctionality of the total T cell response when stimulated with the RSV F_B_ protein peptide library compared to the RSV F_A_ library at all three timepoints (**Figures 4E, F**).

**Figure 5:**
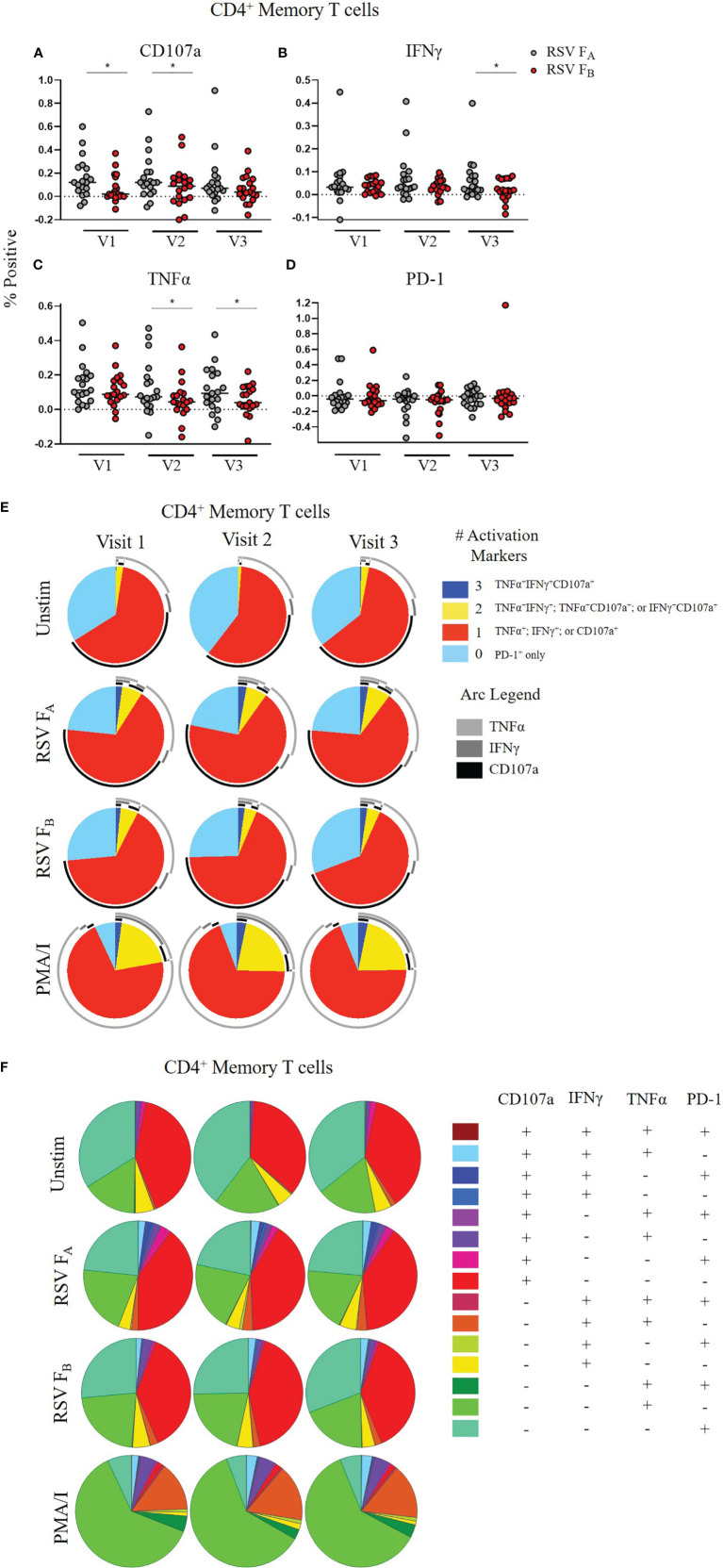
RSV subtype-specific differences in CD4^+^ Memory T cell responses to RSV F protein peptide libraries for all study participants. **(A–D)** Individual functional marker expression. PBMCs were stimulated *in vitro* with RSV F_A_ **(A, B)** or RSV F_B_ **(C, D)** peptide libraries (*n* = 19). Expression of CD107a, IFNγ, TNFα, and PD-1 was measured by flow cytometry and reported as a percentage of CD4^+^ memory T cells. Each symbol represents the response from a single individual. The thick horizontal bar indicates the mean of all responses within each group at that visit. A significant pairwise comparison of mean percentage difference between visits within a group is denoted by a thin horizontal bar with *P ≤ 0.05. V1, Visit 1; V2, Visit 2; V3, Visit 3. **(E)** Polyfunctionality of activation markers in CD4^+^ memory T cell responses by stimulation and study visit. Pie charts represent the mean frequencies of responding CD4^+^CD45RO^+^ T cells following stimulation with RSV F_A_ or RSV F_B_ protein peptide library. Pie charts indicate frequency of cells producing combinations of the activation markers CD107a, IFNγ, and TNFα or expressing PD-1 alone. Background (determined from the media-only negative controls) was subtracted from all samples and negative values were set to zero. **(F)** Total polyfunctionality of CD4^+^ memory T cells by stimulation and study visit. Pie segments indicate frequency of cells producing combinations of all four functional markers CD107a, IFNγ, and TNFα and PD-1. Background (determined from the media-only negative controls) was subtracted from all samples and negative values were set to zero.

**Figure 6:**
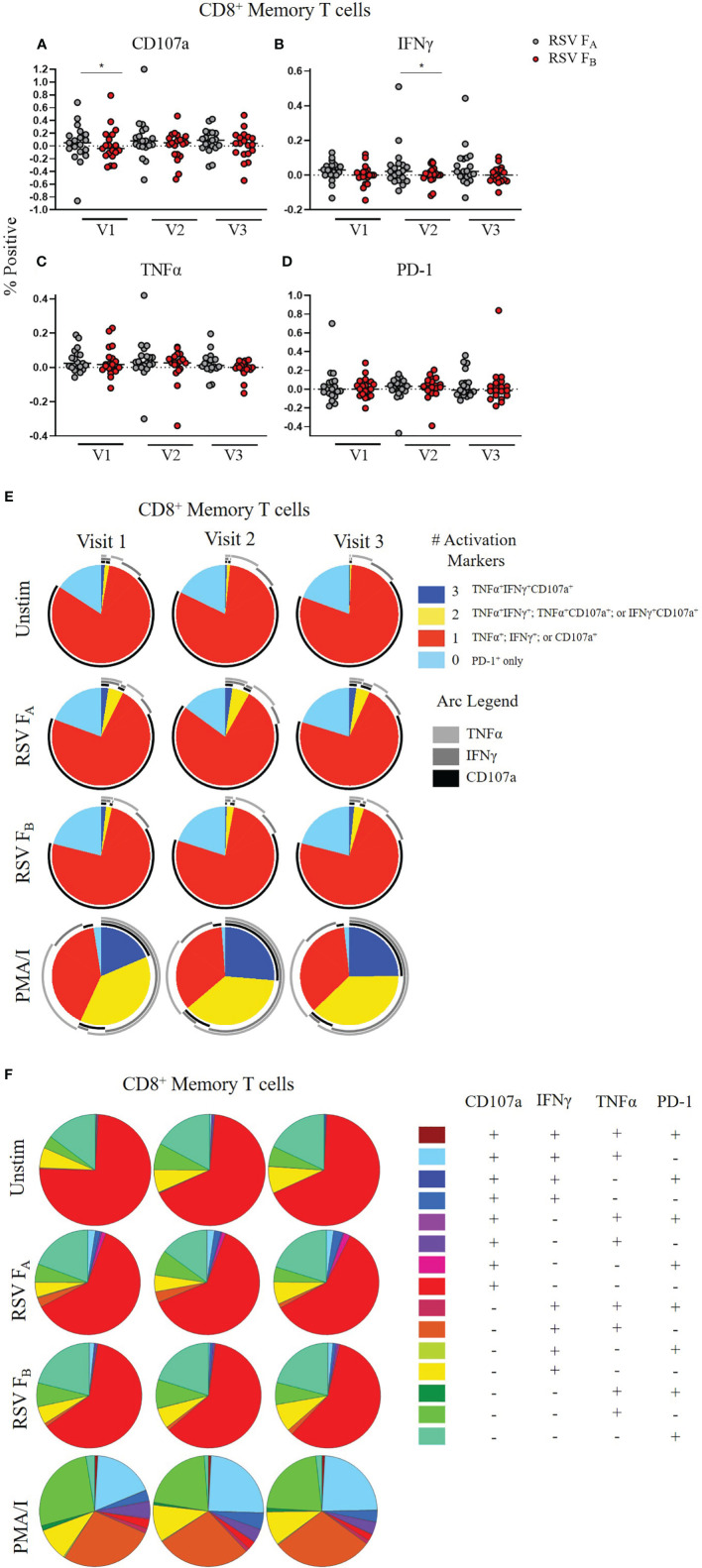
RSV subtype-specific differences in CD8^+^ Memory T cell responses to RSV F protein peptide libraries for all study participants. **(A–D)** Individual functional marker expression. PBMCs were stimulated *in vitro* with RSV F_A_ **(A, B)** or RSV F_B_ **(C, D)** peptide libraries (*n* = 19). Expression of CD107a, IFNγ, TNFα, and PD-1 was measured by ICS and reported as a percentage of CD8^+^ memory T cells. Each symbol represents the response from a single individual. The thick horizontal bar indicates the mean of all responses within each group at that visit. A significant pairwise comparison of mean percentage difference between visits within a group is denoted by a thin horizontal bar with *P ≤ 0.05. V1, Visit 1; V2, Visit 2; V3, Visit 3. **(E)** Polyfunctionality of activation markers in CD8^+^ memory T cell responses by stimulation and study visit. Pie charts represent the mean frequencies of responding CD8^+^CD45RO^+^ T cells following stimulation with RSV F_A_ or RSV F_B_ peptide library. Pie charts indicate frequency of cells producing combinations of the activation markers CD107a, IFNγ, and TNFα or expressing PD-1 alone. Background (determined from the media-only negative controls) was subtracted from all samples and negative values were set to zero. **(F)** Total polyfunctionality of CD8^+^ memory T cells by stimulation and study visit. Pie segments indicate frequency of cells producing combinations of all four functional markers CD107a, IFNγ, and TNFα and PD-1. Background (determined from the media-only negative controls) was subtracted from all samples and negative values were set to zero.

We tested whether a specific compartment of the T cell response is driving these RSV subtype-specific differences. CD4^+^ memory T cells demonstrated significantly higher CD107a, IFNγ, and TNFα expression at most time points when stimulated with RSV F_A_ versus RSV F_B_ protein peptide library (**Figures 5A–D**). There was also a subtle increased polyfunctionality of CD4^+^ memory T cells stimulated with the RSV F_A_ library (**Figures 5E, F**). CD8^+^ memory T cells displayed subtle differences in single marker expression between the two F protein peptide libraries, with significantly higher expression of CD107a at Visit 1 and IFNγ at Visit 2 (**Figures 6A–D**). There was a marked reduction in polyfunctionality of CD8^+^ memory T cells when stimulated with RSV F_B_ protein peptide library compared with the RSV F_A_ protein peptide library (**Figures 6E, F**). Therefore, memory T cells from both major subsets (CD4^+^ and CD8^+^), drive these RSV subtype-specific differences in responses.

### RSV-Subtype Specific Differences in T Cell Responses Are Not Due to HLA-Haplotypes

Although the RSV/A/Ontario and RSV/B/B1 F protein sequences utilized to construct the F protein peptide libraries are very highly conserved (91% sequence homology), even small amino acid changes can lead to alternative T cell epitope recognition by individuals with specific HLA genotypes, which could potentially explain the RSV subtype-related differences in the T cell responses we observed. To test whether these subtype-specific differences are due to alternate epitope recognition originating from the HLA-restriction of the subjects in our cohort, we performed high resolution HLA-typing on all subjects in the study. We then predicted HLA-restricted epitopes within the RSV F protein (RSV/A/Ontario and RSV/B/B1) utilizing MHC class I and class II predictive algorithms (data not shown). We mapped these potential epitopes along the RSV F protein sequences to identify potential epitope ‘hotspots’ within each peptide library (data not shown). We found similar hotspots by subtype where the highest T cell epitope predictions (lowest rank scores) for MHC class II are consistently near the N terminus and between aa 150–250. The list of potential epitopes was refined by utilizing only those contained within both peptide libraries. Both subtypes had similar predicted epitopes within the 15mer peptide libraries (data not shown), indicating the RSV subtype-specific differences in T cell response do not stem from an inability of the adults in our cohort to respond to the peptide libraries because of antigen presentation.

### RSV-Specific T Cell and Neutralizing Antibody Responses Are Correlated

**Figure 7:**
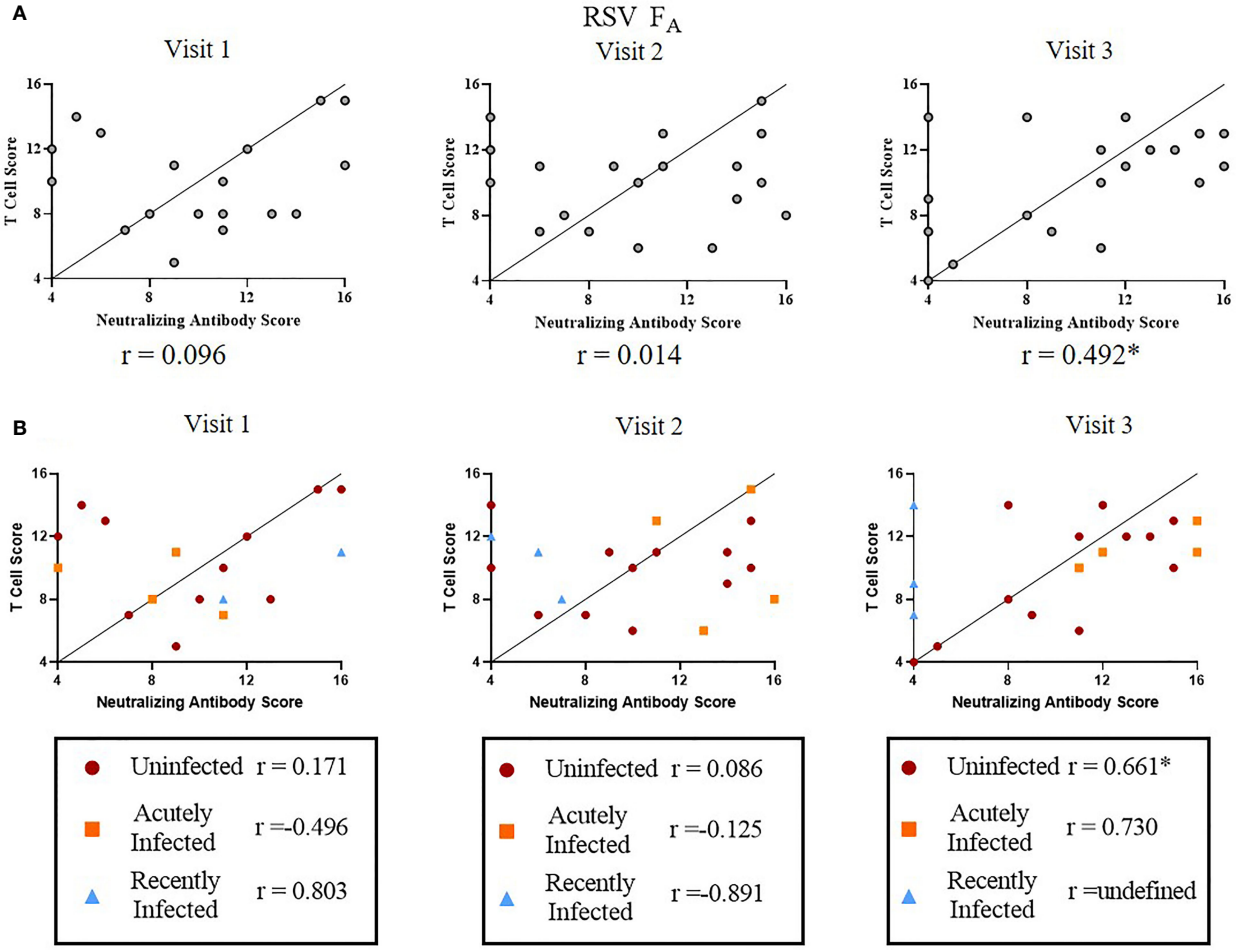
Correlation between RSV F_A_ T cell and neutralizing antibody responses. A significant linear relationship between T cell score and neutralizing antibody score is denoted by a correlation coefficient (r) with *P ≤ 0.05. **(A)** Correlation between RSV F_A_ T cell score with neutralizing antibody score by study visit (*n* = 19). **(B)** Correlation between RSV F_A_ T cell scores with neutralizing antibody score by RSV infection status and study visit. Uninfected (*n* = 12), acutely infected (*n* = 4), or recently infected (*n* = 3) individuals are shown.

Finally, we tested the relationship between T cell and neutralizing antibody responses among RSV infection status or RSV subtype (**Figure 7** and **Supplementary Figure 8**). We were interested in determining if individuals with higher RSV-specific T cell activity also had higher RSV-specific neutralizing antibody levels. We used quartile-ranking of T cell and neutralizing antibody responses to test this hypothesis. We found that T cell and antibody scores were not correlated at Visit 1 or 2 but were highly correlated at Visit 3 for both RSV subtypes (**Figure 7A** and **Supplementary Figure 8A**). We found that the uninfected group was distributed evenly among the quartiles at Visits 1 and 2 (**Figure 7B** and **Supplementary Figure 8B**). At Visit 3 there was a significant correlation of the RSV F_A_ T cell score and neutralizing antibody score in the uninfected group. There are, however, individuals with low quartile scores in the uninfected group, suggesting a small subset of this group may now be susceptible to re-infection. The acutely infected group had scores in the low quartile ranges for both antibody and T cell scores at Visit 1 but, following re-infection, these individuals ended in the high quartiles for both responses, suggesting protection from re-infection. Although not statistically significant, both RSV F_A_ and RSV F_B_ T cell scores nonetheless were highly correlated at Visit 3 (RSV F_A_: *r* = 0.730; RSV F_B_: *r* = 0.880). At enrollment, the recently infected group was in the high quartiles, but dropped to the low quartiles by Visit 2. The correlation coefficient for the recently infected group is undefined at Visit 3, suggesting no relationship between T cell and antibody responses. Therefore, by Visit 3, individuals who were high T cell responders were also high neutralizing antibody responders and those with low T cell responses had low neutralizing antibody responses.

## Discussion

In this study we analyzed the memory T cell response to RSV F protein peptide libraries in a cohort of healthy adults with three distinct antibody kinetic profiles corresponding to their RSV infection status. We found that memory T cell responses mimic previously published antibody responses observed for the three distinct RSV infection status groups (23). Both the acutely and recently infected groups had reduced T cell polyfunctionality compared to the uninfected group at enrollment (Visit 1: early in the RSV season), indicating that higher RSV-specific memory T cell polyfunctionality may protect against re-infection. T cells from the acutely infected group displayed higher PD-1 expression, particularly at enrollment and even without stimulation, suggesting that these individuals’ T cells may have been exhausted prior to infection, which may predispose them to RSV re-infection. Higher expression of PD-1 may also play an inhibitory role during the CD8^+^ T effector cell transition to impair T cell differentiation and subsequent viral clearance during acute infection (38, 39). Additional studies with a larger cohort are warranted to test whether polyfunctionality of memory T cells can be used as a correlate of infection in the adult population.

Increased individual functional marker expression and increased polyfunctionality across all T cell subsets to the RSV F_A_ F protein peptide library rather than the RSV F_B_ protein peptide library was unexpected, as RSV/B was the dominant circulating subtype during the study period (23). Additionally, the highest fold changes in neutralizing antibody were detected to a prototypic B strain (RSV/B/18537), which is analogous to the RSV/B/B1 strain used for generating the F protein peptide library used in this study, and the lowest fold changes were detected to a contemporaneous RSV/A strain (RSV/A/ON) (23). Although the reason for this is unknown, the difference in subtype-specific T cell responses raises several interesting questions. Is this higher T cell response to the RSV F_A_ protein peptide library characteristic of adults in general? If so, do the elderly consistently have a stronger T cell response to RSV/A F protein? Do these lower T cell responses to the RSV F_B_ protein peptide library make adults more susceptible to RSV/B than RSV/A infections? Or is this difference in response reflective of what these particular adults were primed with in prior respiratory seasons? Additional studies testing the T cell responses of older adults, particularly with emphasis on polyfunctionality, as well as the frequency and severity of re-infection by subtype in this population, are warranted. These subtype-related differences have implications for vaccine development, as most vaccine candidates are derived from a single RSV/A strain (prototypic GA1). Our data indicate that adults may need additional protection from RSV/B, so bivalent vaccines containing both RSV subtypes may be warranted, at least for the older adult population.

The recently infected group had a significant decrease in memory T cell single marker expression over the RSV season and marked reduction of polyfunctionality of memory T cells in comparison with the uninfected group at Visit 3, implying these individuals have a lower overall quality of RSV-specific T cell response. Taken together with the antibody response profiles of these individuals (23), these results suggest an overall inability to sustain long-lived memory from both B and T cell responses. The rapid decay of antibody observed in the recently infected group closely resembles the natural decay of immunoglobulin in the absence of newly generated antibody (53, 54). This decay indicates that the antibody response in these individuals could be driven primarily by short-lived circulating plasma blasts that can secrete large amounts of antibody rapidly following infection rather than long-lived plasma cells that typically reside in bone marrow and maintain high levels of antibody long-term (55). Short-lived circulating plasma blasts are typically derived from an extrafollicular response unlike long-lived plasma cells, which are thought to be generated primarily through germinal center responses (55). We hypothesize that individuals in the recently infected group are predisposed to elicit primarily an extrafollicular rather than germinal center response to RSV infection. Predisposition toward an extrafollicular-dominant T cell response may have arisen during the primary exposure in infancy or, more likely, during multiple re-infection events throughout life.

The short-lived antibody response may not be limited to RSV but may hold true for other seasonal respiratory viruses. Indeed, we observed a rapid loss of hMPV-specific antibody responses within this cohort (23). Mechanistic studies aimed at elucidating the underlying cause of these various infection kinetic profiles of long-term memory will have significant impact on vaccine development for respiratory pathogens at large.

Whether or not there is a relationship between the T cell response and a known correlate of protection, neutralizing antibody, is unclear. By the end of our study period, we saw a significant correlation between T cell response scores (to both RSV subtypes) and neutralizing antibody scores. This correlation indicates that individuals with high neutralizing antibody responses are likely to have strong T cell responses (and *vice versa*). It is not surprising that the highest correlation is at Visit 3 compared to earlier study visits, as a limitation of the study is the timing of sample collections to capture the kinetics of T cell responses immediately following infection. As infections were defined using fold-changes in neutralizing antibody rather than PCR, the exact timing of RSV infection in the infected groups is unknown. Therefore, we are best able to detect a relationship at Visit 3, when all subjects have reached a steady-state in their RSV-specific immune response. A relationship between neutralizing antibody and T cell responses suggests that including T cell scores and using them in conjunction with neutralizing antibody responses may strengthen the ability to use them as a correlate of infection and help to identify individuals at higher risk for re-infection. Furthermore, there are differences in the scores by RSV infection status. The two infection groups have opposite patterns in that, the acutely infected group starts within the lowest quartile scores for both T cell and antibody responses, but by the end of the season have the highest for both. The recently infected group starts with high scores for both responses, but by the end of the season there is no relationship between scores. Together, these results strengthen the hypothesis that combined use of T cell scores and neutralizing antibody scores can be used as a correlate of infection.

In summary, we identified three distinct T cell immune responses to the RSV F protein peptide libraries that reflect three distinct antibody kinetic profiles. This increased understanding of how long RSV-subtype specific memory T cell responses persist and how this longevity relates to antibody responses increases our knowledge of how some adults become susceptible to re-infection. This knowledge is vital for developing an efficacious RSV vaccine, particularly in older adult populations where pre-existing immunity may need to be ‘re-trained’ for establishing an optimal and durable immune response upon vaccination.

## Data Availability Statement

The original contributions presented in the study are included in the article/**Supplementary Materials**. Further inquiries can be directed to the corresponding author.

## Ethics Statement

The studies involving human participants were reviewed and approved by Institutional Review Board at Baylor College of Medicine. The patients/participants provided their written informed consent to participate in this study.

## Author Contributions

BNB, PAP, BEG, and LZ designed the study, BNB performed data collection, BNB, LSA, LFS, DH, and PAP planned and conducted data analysis, LSA, VA, LFS, and MC helped with acquisition of data, all authors contributed to interpretation of data and to the decision to publish. BNB completed first and subsequent drafts of the manuscript, and all authors provided feedback and approved the final manuscript.

## Funding

Funding was from discretionary funds from PAP and BEG, and NIH grant R01GM115501 to LZ. This project was further supported by the Cytometry and Cell Sorting Core at Baylor College of Medicine with funding from the CPRIT Core Facility Support Award (CPRIT-RP180672), the NIH (CA125123 and RR024574) and the assistance of Joel M. Sederstrom.

## Conflict of Interest

The authors declare that the research was conducted in the absence of any commercial or financial relationships that could be construed as a potential conflict of interest.

## Publisher’s Note

All claims expressed in this article are solely those of the authors and do not necessarily represent those of their affiliated organizations, or those of the publisher, the editors and the reviewers. Any product that may be evaluated in this article, or claim that may be made by its manufacturer, is not guaranteed or endorsed by the publisher.
