# Supplementary material for: Adult Memory T Cell Responses to the Respiratory Syncytial Virus Fusion Protein During a Single RSV Season (2018–2019)
Source: Front Immunol. 2022 Mar 29;13:823652. doi: 10.3389/fimmu.2022.823652 (PMC9002099; doi:10.3389/fimmu.2022.823652)

**Supplemental Material**

**Supplemental Table 1. Multiparameter flow cytometry antibody panel**

|  | **Fluorophores** | **Markers** | **Clone** | **Company** | **Cat. #** |
| --- | --- | --- | --- | --- | --- |
| **1** | PE/Cy7 | CD45 | HI30 | Biolegend | 304016 |
| **2** | BUV737 | CD3 | UCHT1 | BD | 564307 |
| **3** | AF488 | CD4 | RPA-T4 | Biolegend | 300519 |
| **4** | BV605 | CD8 | RPA-T8 | Biolegend | 301040 |
| **5** | BV421 | CD45RO | UCHL1 | Biolegend | 304224 |
| **6** | BV785 | CD107a | H4A3 | Biolegend | 328644 |
| **7** | BV510 | TNF-α | MAb11 | Biolegend | 502950 |
| **8** | AF700 | IFN-γ | 4S.B3 | Biolegend | 502520 |
| **9** | APC/Fire750 | PD-1 | EH12.2H7 | Biolegend | 329954 |
| **10** | PE | CD56 | HCD56 | Biolegend | 318306 |
| **11** | APC | CD16 | 3G8 | Biolegend | 302012 |
| **12** | UV450 | Viability | LIVE/DEAD® Fixable Blue Dead Cell Stain Kit, for UV excitation | LifeTechnologies | L34961 |


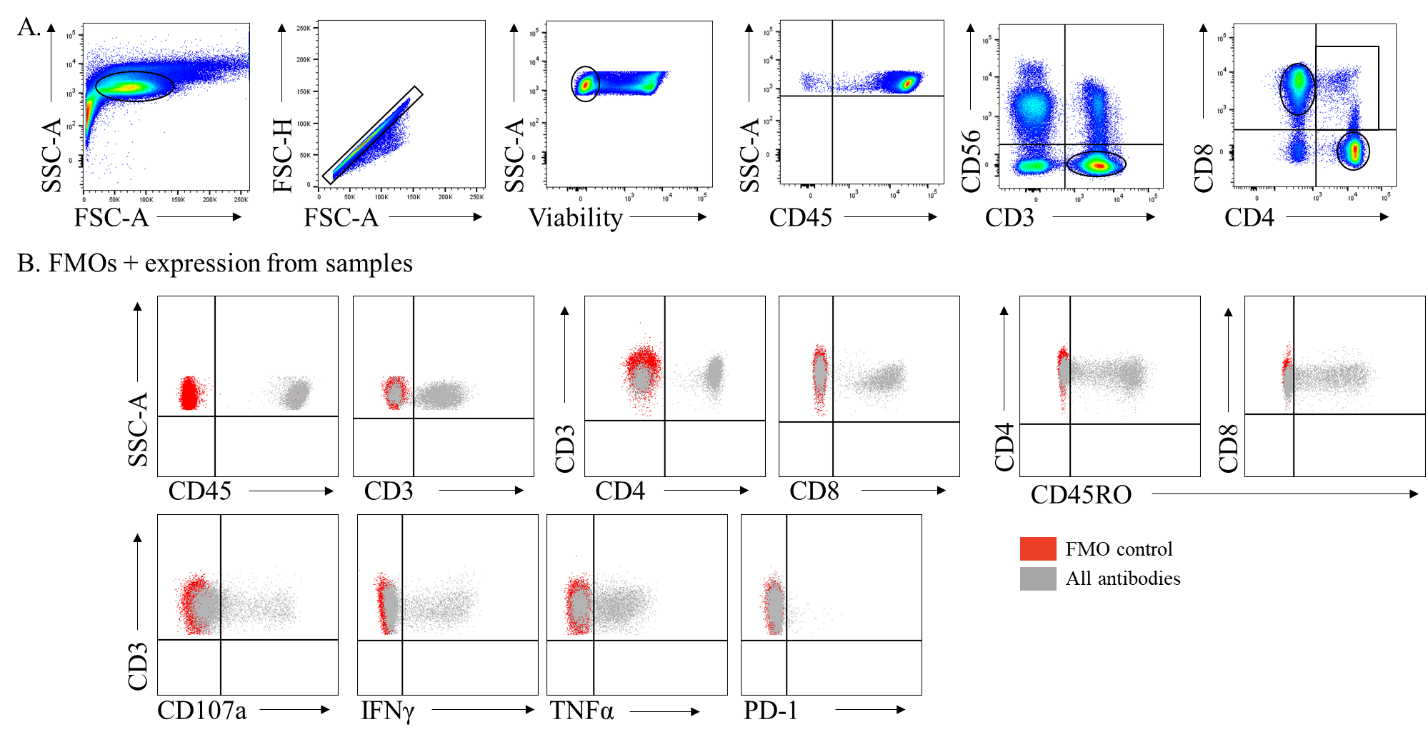
**Supplemental Figure 1.** Representative gating strategy. **A** T Lymphocytes were distinguished by FSC-A and SSC-A. Single, live cells were identified, and lymphocytes further defined by CD45 expression. CD3 and CD56 were analyzed simultaneously to exclude NK and NKT cells from further analysis. CD4, CD8, or CD4/CD8 T cells were then identified. Memory subsets were defined by CD45RO^+^ expression. **B** Representative example of marker expression overlayed with FMO control samples. Representative expression of CD107a, IFNγ, TNFα, and PD-1 is from PMA/Ionomycin treated positive controls. Samples were gated using FMO gates from like stimulation conditions.

**Supplemental Figure 2. Total polyfunctionality of total T cells against RSV F_A_ protein peptide library by RSV infection status and study visit.** Peripheral blood mononuclear cells (PBMCs) were stimulated with the RSV F_A_ protein peptide library. Pie charts show the frequency of cells producing the various combinations of the four tested functional markers CD107a, IFNγ, and TNFα and PD-1. Background (determined from the media-only negative controls) was subtracted from all samples and negative values were set to zero. Representative negative (Unstim) and positive controls (PMA/I) across all study visits are boxed.

**
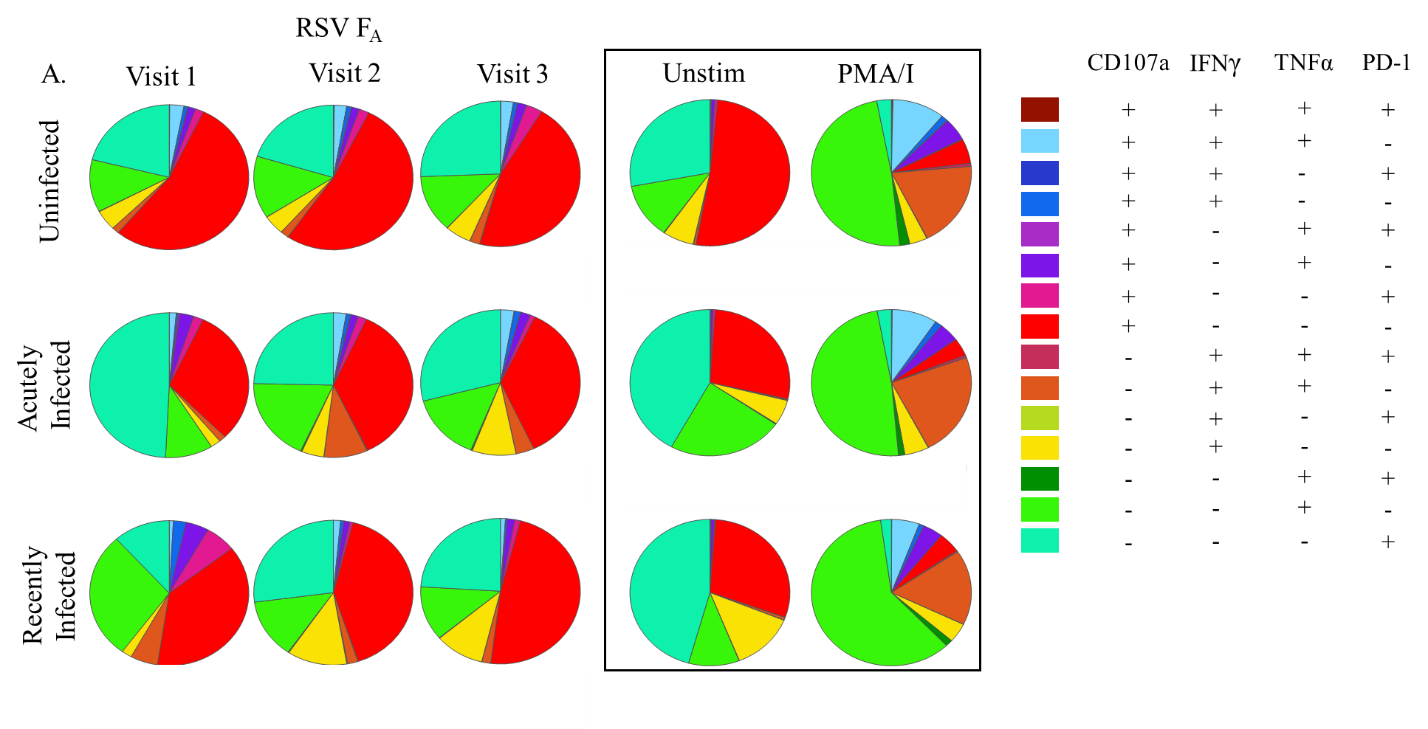
**

**Supplemental Figure 3.** Uniform manifold approximation and projection analysis of T responses to RSV F_A_ and RSV F_B_ protein peptide libraries. **A** Contour plot of total concatenated samples. **B** Overlay of individual marker expression. Scaled expression of each phenotypic marker is overlayed onto the contour plot of total concatenated samples to aid in visual exploration of the data set. **C** Density plots representing 90% of the total gated cells by RSV infection status at each study visit were superimposed upon uniform manifold approximation and projection (UMAP) projections to visualize differences. **D** Density plots representing 90% of the total gated cells by F protein peptide library stimulation and study visit were superimposed upon UMAP projections to visualize differences.

**
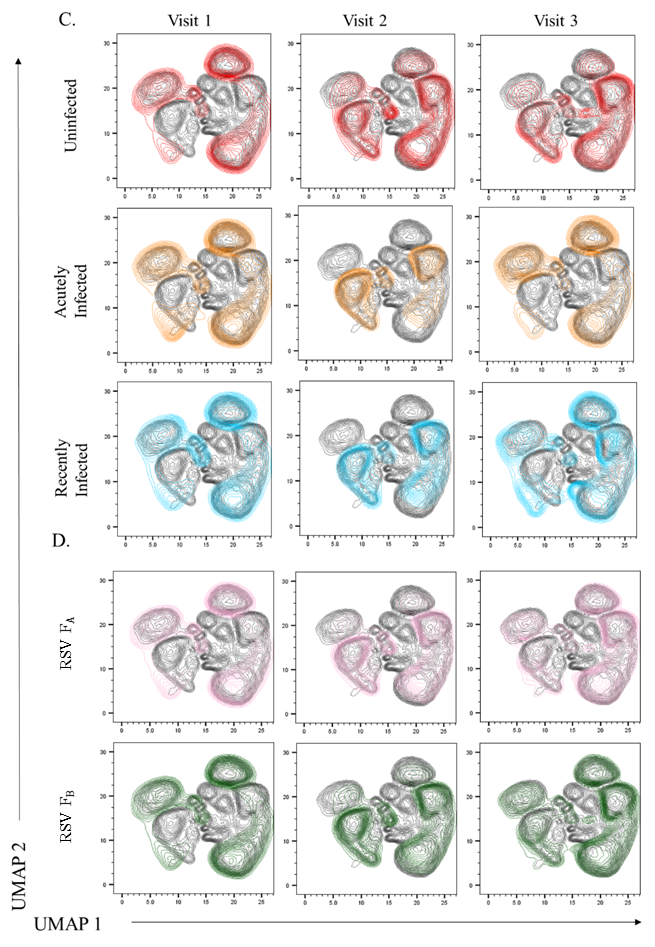

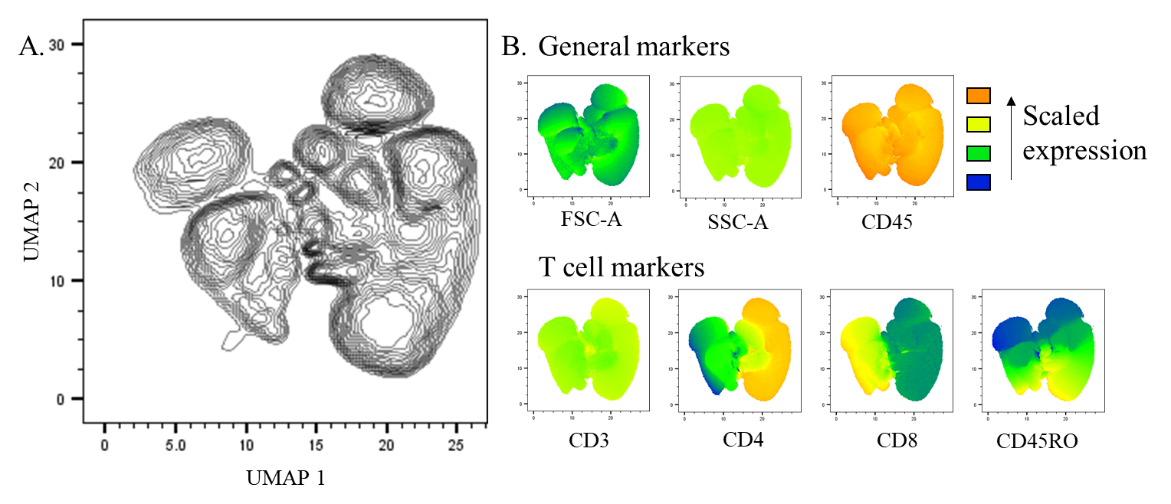
**

**
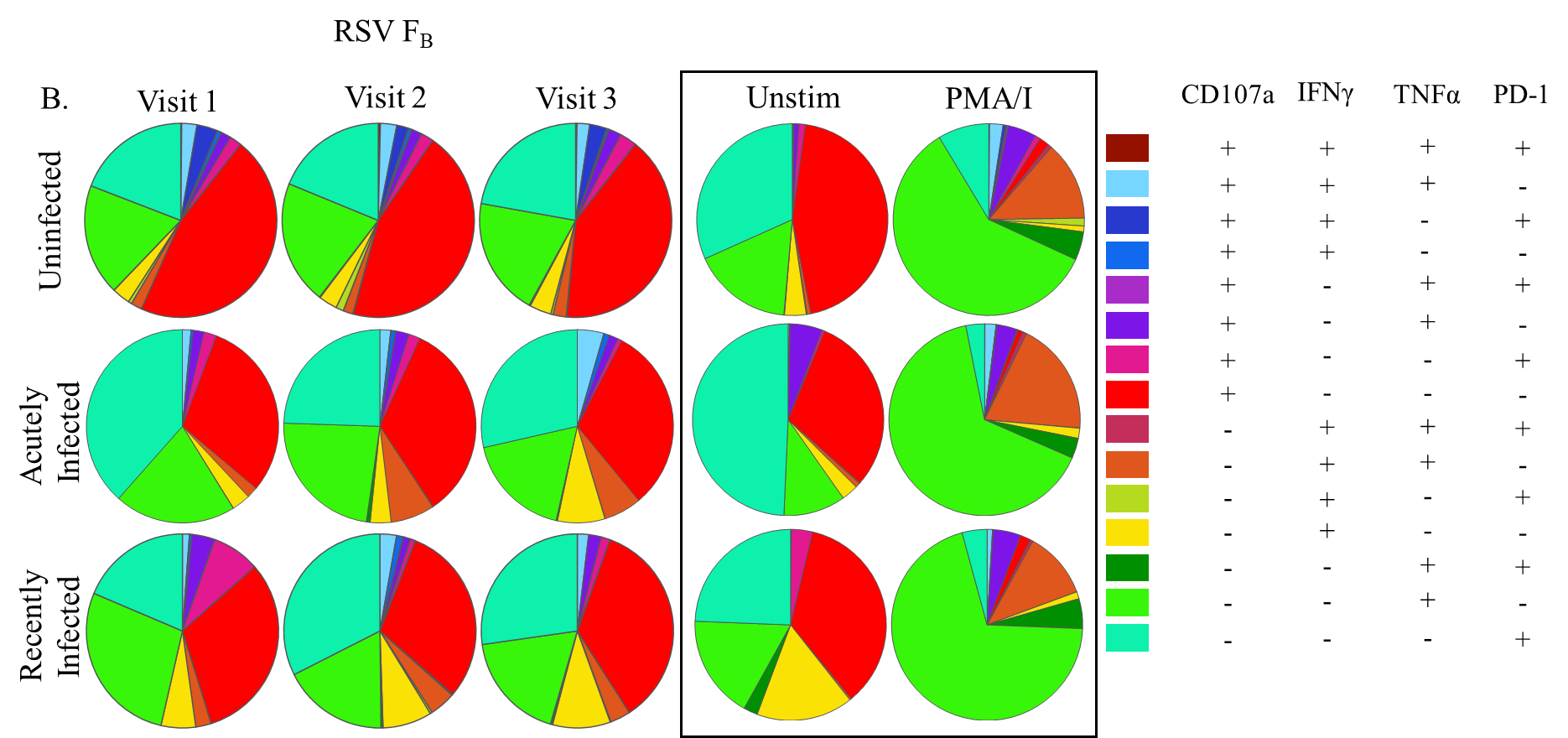

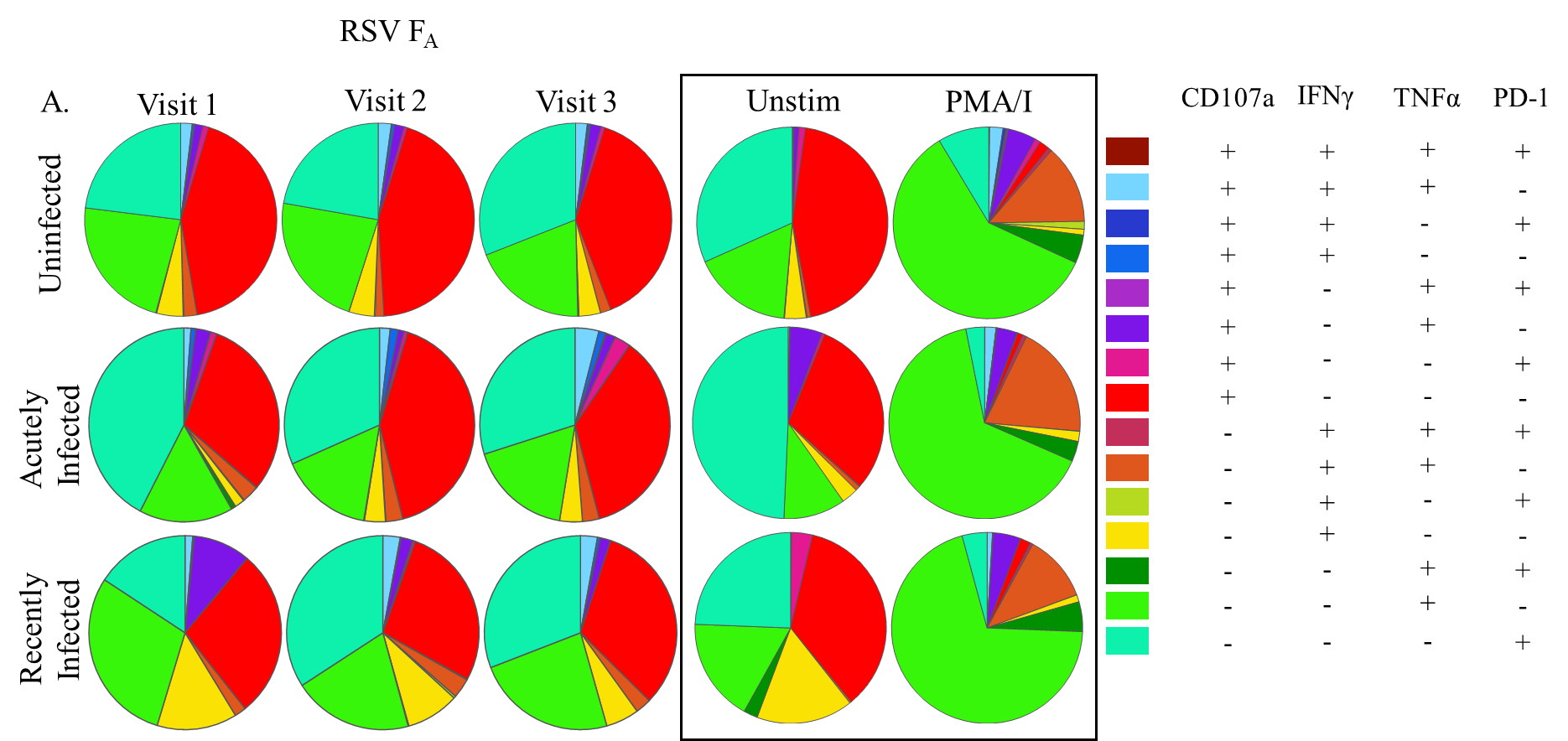
Supplemental Figure 4.** Total polyfunctionality of CD4^+^ Memory T cells against RSV F protein peptide libraries by RSV infection status and study visit. PBMCs were stimulated *in vitro* with **A** RSV F_A_ or **B** RSV F_B_ protein peptide libraries. Pie charts show the frequency of cells producing each of the possible combinations of the functional markers CD107a, IFNγ, and TNFα and PD-1. Background (determined from the media-only negative controls) was subtracted from all samples and negative values were set to zero. Representative negative (Unstim) and positive (PMA/I) controls across all study visits are boxed.

**Supplemental Figure 5.** CD8^+^ Memory T cell individual functional marker response to RSV F protein peptide libraries by RSV infection status: uninfected (*n* = 12), acutely infected (*n* = 4), and recently infected (*n* = 3) and study visit. PBMCs were stimulated with RSV F_A_ or RSV F_B_ protein peptide libraries and expression of CD107a, IFNγ, TNFα, and PD-1 was measured by ICS and reported as percent positive of CD8^+^ memory T cells. Each symbol represents the response from a single individual. The thick horizontal bar indicates the mean of all responses within each group at that visit. A significant pairwise comparison of mean percentage difference between visits within a group is denoted by a thin horizontal bar with **P* ≤ 0.05.

**
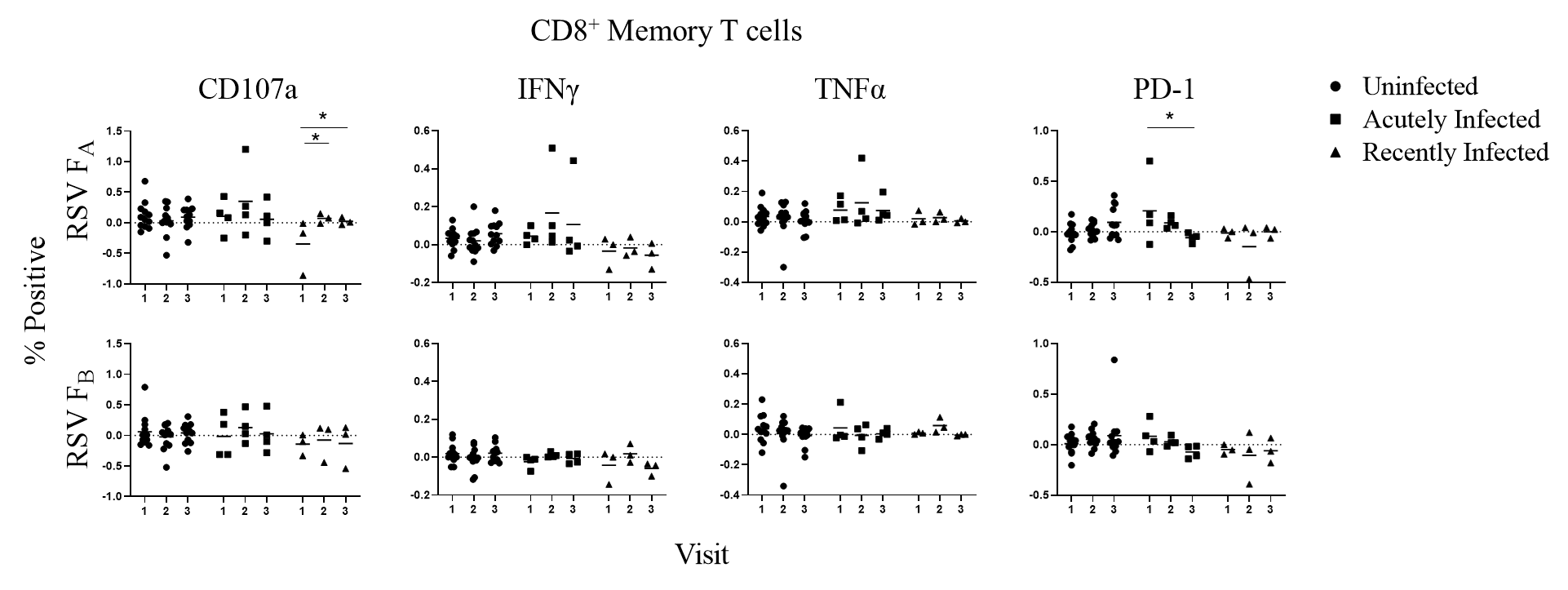
**

**
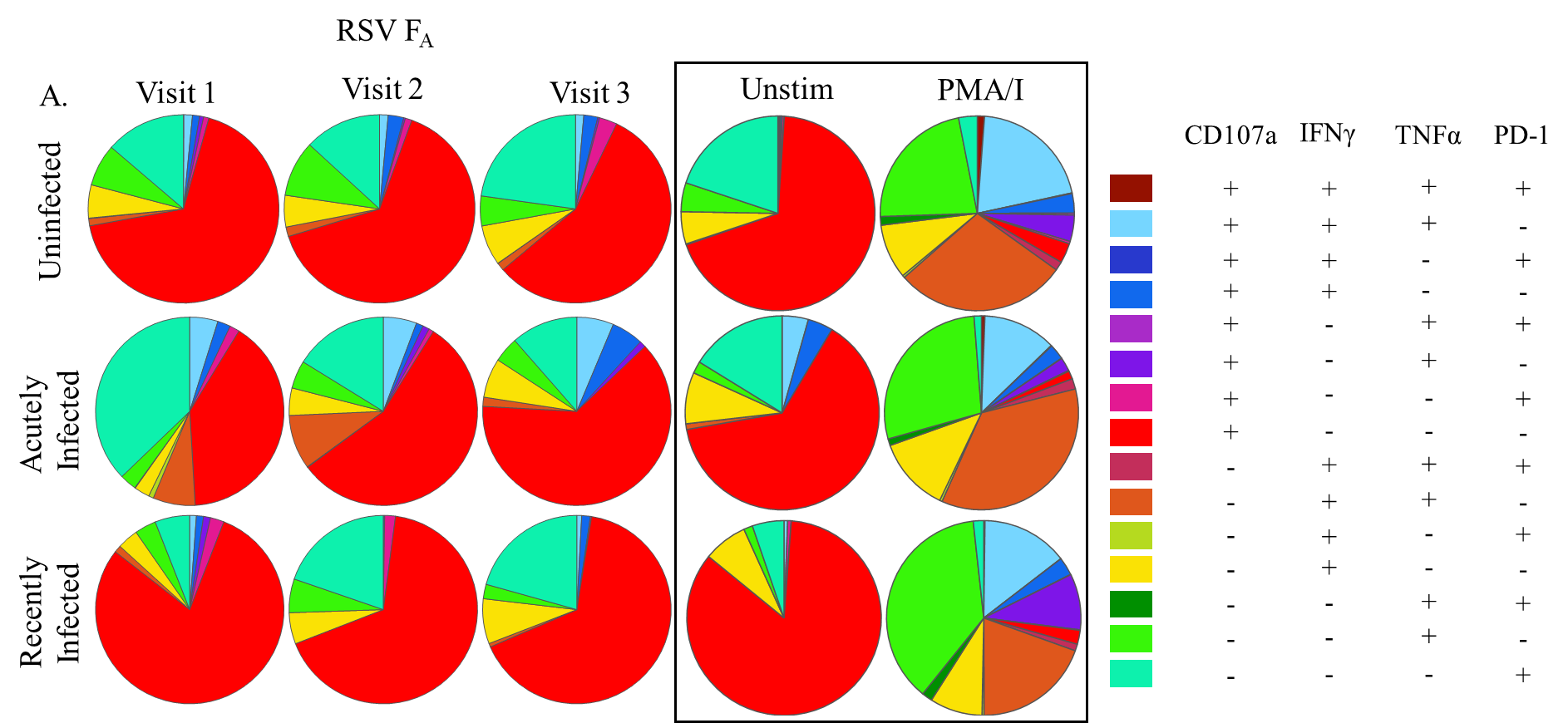
Supplemental Figure 6.** Total polyfunctionality of CD8^+^ Memory T cells against RSV F protein peptide libraries by RSV infection status and study visit. PBMCs were stimulated with **A** RSV F_A_ or **B** RSV F_B_ protein peptide libraries. Pie charts show the frequency of cells producing the indicated combinations of the four functional markers. Background (determined from the media-only negative controls) was subtracted from all samples and negative values were set to zero. Representative negative (Unstim) and positive (PMA/I) controls across all study visits are boxed.

**
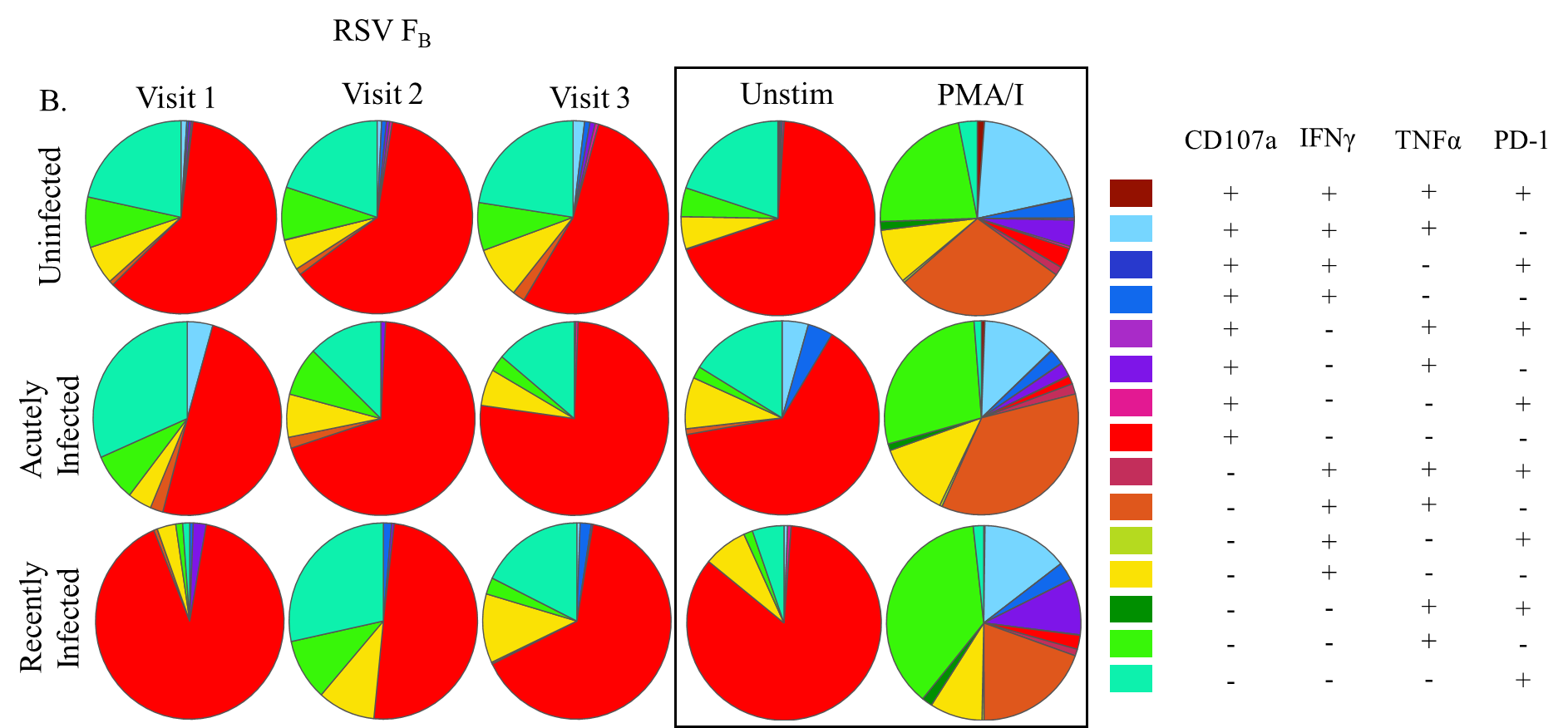
**

**Supplemental Figure 7.** CD4^+^CD8^+^ Memory T cell individual functional marker response to RSV F protein peptide libraries by RSV infection status: uninfected (*n* = 12), acutely infected (*n* = 4), and recently infected (*n* = 3) and study visit. PBMCs were stimulated with RSV F_A_ or RSV F_B_ protein peptide libraries and expression of CD107a and TNFα was measured by ICS and reported as a percentage of CD45RO^+^ CD4^+^CD8^+^ T cells. Each symbol represents the response from a single individual. The thick horizontal bar indicates the mean of all responses within each group at that visit. A significant pairwise comparison of mean percentage difference between visits within a group is denoted by a thin horizontal bar with **P* ≤ 0.05 or ***P* ≤ 0.01.


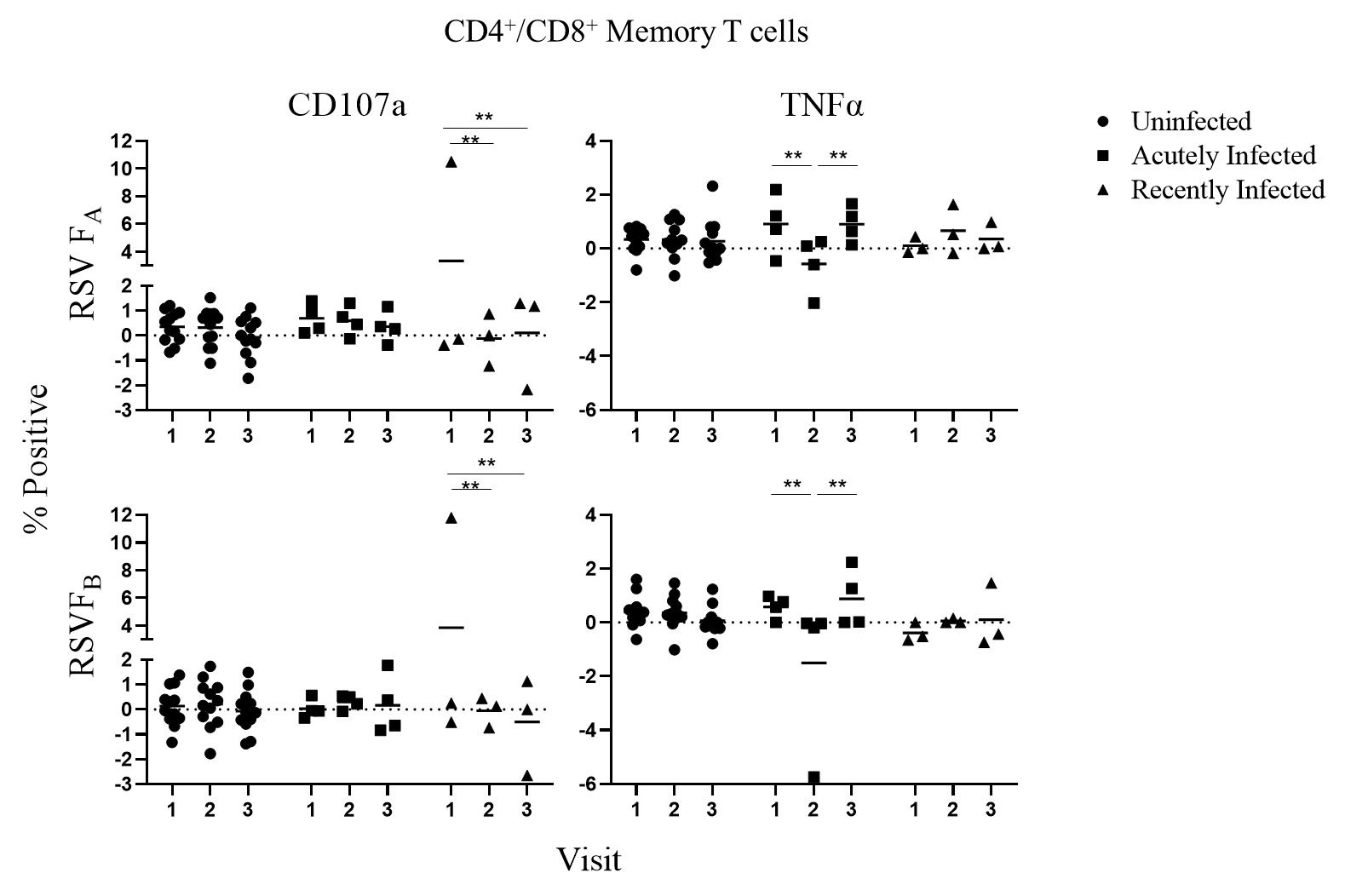


**Supplemental Figure 8.** Correlation between RSV F_B_ T cell and neutralizing antibody responses. **A** Correlation between RSV F_B_ T cell score with neutralizing antibody score by volunteer (*n* = 19) study visit. **B** Correlation between RSV F_B_ T cell scores with neutralizing antibody score by RSV infection status and study visit. Uninfected (*n* = 12), acutely infected (*n* = 4), or recently infected (*n* = 3) individuals are shown.


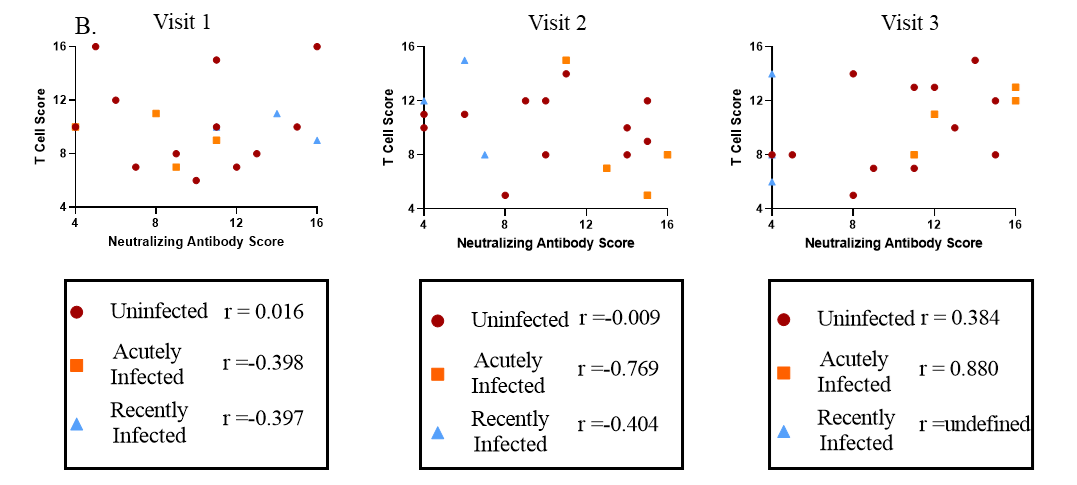

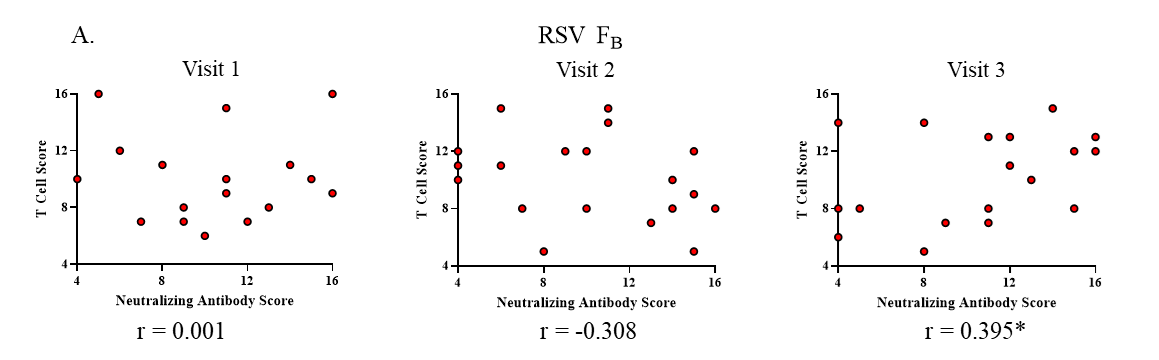

Supplement: Supplementary file 1 [file DataSheet_1.docx]
